# Sir Arthur Newsholme's Annual Report, 1916-17

**Published:** 1917-12-08

**Authors:** 


### THE NATION'S HEALTH.

### Sir Arthur Newsholme's Annual Report, 1916-17.

Few, if any, Governmental publications contain so much information of intense interest to all those who have the welfare of the nation at heart as this unassuming pamphlet, issued under the auspices of the Local Government Board, containing the Report of its medical officer. In it are recorded, frankly and fully, the results of the year's work in communistic hygiene, the fruits of tireless investigation by experts commissioned by the Board to inquire into the causes and prevention of epidemic disease as evidenced by vital statistics. Handicapped as they have been by the exceptional conditions now prevailing, their record is a fine one, deserving of all praise. Under the able direction of Sir Arthur Newsholme, the Department has succeeded not only in carrying on the work of previous years, but in organising additional Services—such, for example, as the new Venereal Clinics—of paramount importance to the nation, with conspicuous success. It is obviously impossible within the scope of a brief article to do more than touch upon the principal points dealt with. It is satisfactory to note that the fears entertained of widespread epidemics caused by the return of soldiers from the Front have proved groundless. There have been no cases of cholera, and the few cases of plague which occurred were brought rapidly under control. Typhus has failed to spread both in the civil and military population, and the amount of enteric has been exceptionally small. Cerebro-spinal fever, as a result of the investigations undertaken upon it, has been less active than in 1915, there being 1,306 cases notified to 2,566 in the previous year.

### Tuberculosis Work in 1916.

With regard to tuberculosis the outlook is less satisfactory. There has been much difficulty in persuading county areas and boroughs to complete schemes for institutional treatment, and in those cases where schemes have been made they have usually been in operation only a short time, and have been crippled more or less during 1915 and 1916. The urgent need of the moment seems to be a closer co-operation between the doctor and the tuberculosis officer. Many cases are not notified till they are within a few weeks or months of death, either through failure on the part of the patient to seek medical advice, or because the doctor is so overworked that he has no time to make an adequate examination. The presence of tubercle bacilli in the sputum usually implies that the disease has made considerable progress, and in view of the extreme difficulty of forming an early diagnosis the aid of the specialist ought to be enlisted in all doubtful cases. "The new Service has been regarded in some areas with suspicion by medical practitioners," says the Report, "and little use has been made of the facilities for consultation with the tuberculosis officer." This is perhaps explicable by the fact that at first there was a dearth of men possessing the necessary experience of the diagnosis and treatment of tuberculosis on modern lines. One of the most urgent reforms after the war will be the proper training of men for this work. At present pulmonary tuberculosis is responsible for one-third of the total mortality during a large part of the working years of life; it is moreover a disease that is preventable. One of the greatest difficulties at the present time is the home treatment of insured patients. The majority of insured consumptives could, under proper home conditions, be as well treated at their own dwellings as in an institution. For reasons of economy, a large proportion of the infective life of every patient must necessarily be spent at home, and with proper domiciliary inspection, especially if the patient has spent some months in a sanatorium and has learnt how to attend to himself, there is no reason why he should be a source of danger to others or retard his own cure. But the housing conditions of the poorer classes are such as gravely to militate against the success of home treatment. At the last census, 3,139,472 persons, or 8.7 per cent. of the total population, were living in dwellings containing less than one room for every two persons, there being an excess of 780,776 persons in these dwellings on this basis. Of the total population, 2,580,814, or 7.1 per cent., were living in one- or two-roomed dwellings, and 4,429,119, or 12.3 per cent., in three-roomed dwellings. Under these circumstances the impracticability of providing adequate, and still more of providing separate, bedroom accommodation for consumptive patients is evident. There is no possibility of their being continuously treated in an institution owing to lack of accommodation, nor would there be any need for it were the housing conditions such as to make it possible for them to carry out the simple instructions in which they have been previously educated. At present the total sum spent by the Government on institutions, in grants to local authorities and contributions to insured persons, amounts to less than half a million per annum.

### Saving the Children.

Turning to infant welfare and maternity work, we find an increasing need for reducing our excessive infantile mortality in the markedly lower natural increase in the population. In 1914 there were 362,354 more births than deaths in England and Wales; in 1915 this figure had fallen to 252,201. In 1916 the corresponding excess was 277,227, there being 29,073 fewer births and 54,099 fewer deaths than in 1915. Last year, however, the infantile mortality rate was the lowest on record, 91 per 1,000 registered births; in the great towns it was 99, in the smaller towns 90, and in London only 89. This was in part due, says the Report, to the cool summer, which materially lessened the mortality from infantile diarrhœa, but is also in part due to the direct work which has been carried on to reduce infant mortality. A grant of

**THE NATION'S HEALTH**—(*continued*).

50 per cent. has been allowed by the Board towards defraying the expenses of the local authorities' child welfare schemes, as compared with the 75 per cent. allowed for the treatment of venereal diseases, with the result that the work done has been proportionately less in relation to the total needs. The Report adds: "At the present time fifty-one County Councils have provided for the visiting of women and children by health visitors, forty-one of these schemes covering the whole county, with the exception of sanitary districts having separate schemes. In ten other counties the County Council have provided for a portion of the county area. Of the remaining eleven counties, several are on the point of setting into operation schemes that they have prepared. All the metropolitan boroughs with the exception of Camberwell, and all the county boroughs with the exception of Gateshead, have made some, though often very inadequate, provision for this work, and 360 of the smaller sanitary authorities. There are now 396 centres for maternity and infant welfare work established by local authorities, and 446 by voluntary societies; a large number of the latter receive assistance, financial and otherwise, from the local authorities." The progress of the work in this and other departments has been hampered by the shortage of medical officers. More than 500 public health officers have joined the Forces, and during the year an increasing number of women practitioners have received official appointments. Two are now acting as county medical officers of health, and many as tuberculosis officers and assistant tuberculosis officers. "The depletion of the Public Health Services has become serious, and, unless considerable adjustments are made, more officers cannot be spared consistently with the public safety."

### Maternal Mortality.

Dr. Lane-Claypon, in a special report on maternal mortality in child-birth, emphasises the favourable position of London in regard to puerperal sepsis as compared to the provinces. In counties and county boroughs the rate was 64 per cent. higher than in the Metropolis, this being no doubt due to the better provision in London available for cases of complicated labour. Of a total of 101,649 births in the County of London, 32,461 were attended in their own homes by midwives, an average of 86.1 cases to each midwife. Such a large number of cases per individual did not leave sufficient time to the midwife to devote adequate attention to the woman during the lying-in period, and consequently the patients were often left in the hands of untrained women, so-called monthly nurses, or even to a neighbour's care. When doctors attended confinements the lack of the necessary subsequent care was even more marked, and the provision of more trained monthly nurses was a pressing matter. The earnings of midwives, especially in rural areas, where the number of births is small and the wages low, were inadequate, and should be increased in some way.

### Treatment of Venereal Diseases.

The Report of the Royal Commission on Venereal Diseases was issued in March 1916, and the Board's Regulations, issued in July, gave effect to its most important recommendations, making it obligatory on County Councils and Boroughs to prepare schemes for the provision of the pathological aids to diagnosis, institutional treatment, and the free provision of arseno-benzol preparations for their domiciliary treatment. By the end of March 1917 schemes had been submitted to the Board by 86 out of the 145 Councils charged with execution of the Order, comprising a total of 23,500,000 of the population. Approval had been given to forty-five of these schemes, serving a population of 16,000,000, and the work had been already started at thirty hospitals, whilst between 130 and 140 in England and Wales had expressed their willingness to participate in the schemes of local authorities. Some hospital boards showed unwillingness to undertake the work, either because their rules forbade it, or because they feared if they did so their subscriptions would fall off. In other instances the hospital boards showed an exaggerated fear of the infectiousness of the disease, and demanded the provision of special in- and out-patient departments, and even of special accommodation for the nursing staff. In most cases, however, it has been found possible to utilise the general hospital of the district as a treatment centre, though in some instances patients have to travel long distances for treatment, their travelling expenses being defrayed by the grant. For instance, patients in Westmorland will rely on the Royal Infirmary, Manchester. The treatment of gonorrhœa does not compare in efficacy with that for syphilis, and it is hoped to render available for civilian practitioners the valuable experience secured by Army doctors in its treatment. On the whole it may be said that the scheme is working well under conditions of exceptional difficulty.

